# MicroRNA Transcriptomic Analysis of Heterosis during Maize Seed Germination

**DOI:** 10.1371/journal.pone.0039578

**Published:** 2012-06-27

**Authors:** Dong Ding, Yinju Wang, Mingshui Han, Zhiyuan Fu, Weihua Li, Zonghua Liu, Yanmin Hu, Jihua Tang

**Affiliations:** College of Agronomy, Henan Agricultural University, Zhengzhou, China; University of New England, Australia

## Abstract

Heterosis has been utilized widely in the breeding of maize and other crops, and plays an important role in increasing yield, improving quality and enhancing stresses resistance, but the molecular mechanism responsible for heterosis is far from clear. To illustrate whether miRNA-dependent gene regulation is responsible for heterosis during maize germination, a deep-sequencing technique was applied to germinating embryos of a maize hybrid, Yuyu22, which is cultivated widely in China and its parental inbred lines, Yu87-1 and Zong3. The target genes of several miRNAs showing significant expression in the hybrid and parental lines were predicted and tested using real-time PCR. A total of 107 conserved maize miRNAs were co-detected in the hybrid and parental lines. Most of these miRNAs were expressed non-additively in the hybrid compared to its parental lines. These results indicated that miRNAs might participate in heterosis during maize germination and exert an influence via the decay of their target genes. Novel miRNAs were predicted follow a rigorous criterion and only the miRNAs detected in all three samples were treated as a novel maize miRNA. In total, 34 miRNAs belonged to 20 miRNA families were predicted in germinating maize seeds. Global repression of miRNAs in the hybrid, which might result in enhanced gene expression, might be one reason why the hybrid showed higher embryo germination vigor compared to its parental lines.

## Introduction

Heterosis is defined as the superior performance of F_1_ hybrids compared to their parental inbred lines [1]. The phenomenon can be manifested in many phenotypes such as strong growth vigor, increased plant height, and high yield. Utilization of heterosis in many agronomically important crops, such as maize and rice, provides higher yields than pure lines; therefore many researchers have sought to elucidate the molecular mechanism of heterosis. Three major hypotheses have been proposed to explain the genetic basis of heterosis, namely the dominance [2], overdominance [3], and epistasis hypotheses [4].

Maize is a suitable model for exploration of the genetic mechanism of heterosis, because it contains high levels of phenotypic, allelic [5], transcriptional [6]–[7], and translational [8] variation and the ready availability of maize genomic information. In maize, heterosis-related QTLs have been mapped onto the genome, but this technique relies on marker-assisted selection [9], and heterosis-related transcripts and proteins have been captured by high-throughput genome sequencing and 2-DE analysis [10]–[11]. However, because of its complex character, the genetic mechanism underlying heterosis in maize is far from clearly understood. The main difficulty is that manifestation of heterosis reflects environmentally modified quantitative phenotypes, caused by complex molecular events and gene interactions, so a major gene or gene pathway alone is unlikely to represent the genetic basis of heterosis in maize.

MicroRNAs (miRNAs) are approximately 21-nucleotide non-coding RNAs that play critical roles in the regulation of gene expression at the post-transcriptional level, and are especially important in epigenetic gene regulation. In plants, cleavage of the target mRNA appears to be the predominant method of post-transcriptional regulation, yet plant miRNA-guided gene silencing has a widespread translational inhibitory component [12]. Plant miRNA-guided gene regulation is involved in multiple developmental processes, such as organ polarity [13], leaf growth [14], sex determination [15], and male or female sterility [16]. One maize miRNA, miR167, was over dominantly induced in samples of kernels 10 d after pollination between H99 × B73 and its parental inbred lines, which led to the suggestion that miRNAs might be involved in the regulation of heterosis-related genes [17].

The best starting point for exploration of the genetic mechanism of heterosis at the molecular level is to focus on a simple trait that is rarely influenced by environmental variability or other traits [18]. Recently, it was reported that maize embryos soaked for 24 h show heterotic behavior [11], and that distinct difference in germination vigor between hybrids and their parental inbred lines were exhibited; the embryonic tissues in this state are barely influenced by dosage-related effects from the triploid endosperm and environmental effects. In this study, miRNAs in embryos of a maize hybrid, Yuyu22, and its parental inbred lines soaked for 24 h were deep-sequenced to investigate the involvement of miRNAs in the heterosis of germinating embryos and to gain insights into regulatory pathways governing gene expression.

## Results

### MicroRNAs Detected in the Maize Hybrid and Parental Lines

The miRNAs detected in the maize hybrid Yuyu22 and its parental inbred lines, Yu87-1 and Zong3, were deep-sequenced using Solexa technology. All of the miRNAs detected were compared to maize miRNAs in the miRBase database (http://www.mirbase.org/, Version 18.0; 171 mature miRNAs contained) [19]. A total of 126 miRNAs from Yuyu22, 122 small RNAs from Yu87-1, and 130 small RNAs from Zong3 were matched to known miRNAs (Table S1). In total, 107 known miRNAs belonging to 21 miRNA families were co-detected in the hybrid and its two parental lines; one miRNA was specifically detected in Yu87-1, twelve were specific to Zong3, and three were detected only in the hybrid. The codetected miRNAs might be present in all maize lines and the unique miRNAs might be hybrid-specific or inbred line-specific.

### Expression Pattern of the microRNAs in the Hybrid and Parental Lines

The 107 co-detected miRNAs and their detected reads per million (RPM) in each line are shown in Table S1. The expression trends could be explained by the additive, dominance, and over-dominance hypotheses. Most (94/107) of the miRNAs were differentially (non-additively) expressed in the germinating embryos between the hybrid and the two inbred lines. The dominance influence should discriminate between maternal or paternal dominance (higher or lower expression level in the parental line), and over-dominance was manifested as up- or down-regulation of expression, thus the co-detected miRNAs were classified into six groups (Table 1). The most abundant miRNA group was over-dominant down-regulated (–, the difference of expression amounts between hybrid and low expression parental line was beyond the threshold 10%) miRNAs, which comprised 37 miRNA members from 14 miRNA families, followed by 28 miRNA members from four miRNA families that were dominant with low expression in the parental line (-, the difference of expression amounts between hybrid and low expression parental line was below the threshold 10%). Thirteen miRNA members from four miRNA families were additively expressed (+-,the difference of expression amounts between hybrid and mid-parental values was below the threshold 10%),four miRNA members from the miR167 family were overdominant and up-regulated (++, the difference of expression amounts between hybrid and high expression parental line was beyond the threshold 10%), and six miRNA members from the miR171 family were dominant with high expression in the parental line (+, the difference of expression amounts between hybrid and high expression parental line was below the threshold 10%), and 20 miRNA members from seven miRNA families showed different expression patterns distinct from all of the above classes (D, the difference of the expression amounts between hybrid and parental lines and the difference between hybrid and mid-parental value were both indefinable when using 10% as the threshold). It is interesting that about two-thirds (14/21) of the miRNA families were repressed (– and -) in the hybrid compared to the parental inbred lines.

**Table 1:** Non-additively expressed miRNAs in the maize hybrid Yuyu22 and its parental inbred lines.

	Non-additive miRNA type						Total number of non-additive miRNAs
	+ +^a^	+^b^	+ −c	D^d^	−e	− −f	
miRNA families	1	1	4	7	4	14	21
miRNA members	4	6	13	20	28	37	107*

Note:^ a^ + +: Extremely high parental expression, the difference of expression amounts between hybrid and high expression parental line was beyond the threshold 10%;

b +: High parental expression, the difference of expression amounts between hybrid and high expression parental line was below the threshold 10%;

c + −: additive expression, the difference of expression amounts between hybrid and mid-parental values was below the threshold 10%;

d D: Different from additivity (mid-parent value), not belonging to any of the other classes, the difference of the expression amounts between hybrid and parental lines and the difference between hybrid and mid-parental value were both indefinable when using 10% as the threshold;

e −: Low parental expression, the difference of expression amounts between hybrid and low expression parental line was below the threshold 10%;

f − −: Extremely low parental expression, the difference of expression amounts between hybrid and low expression parental line was beyond the threshold 10%;

* : miR156k was defined both as + − and −.

### Novel Maize miRNAs Derived from Deep-sequencing

**Figure 1:**
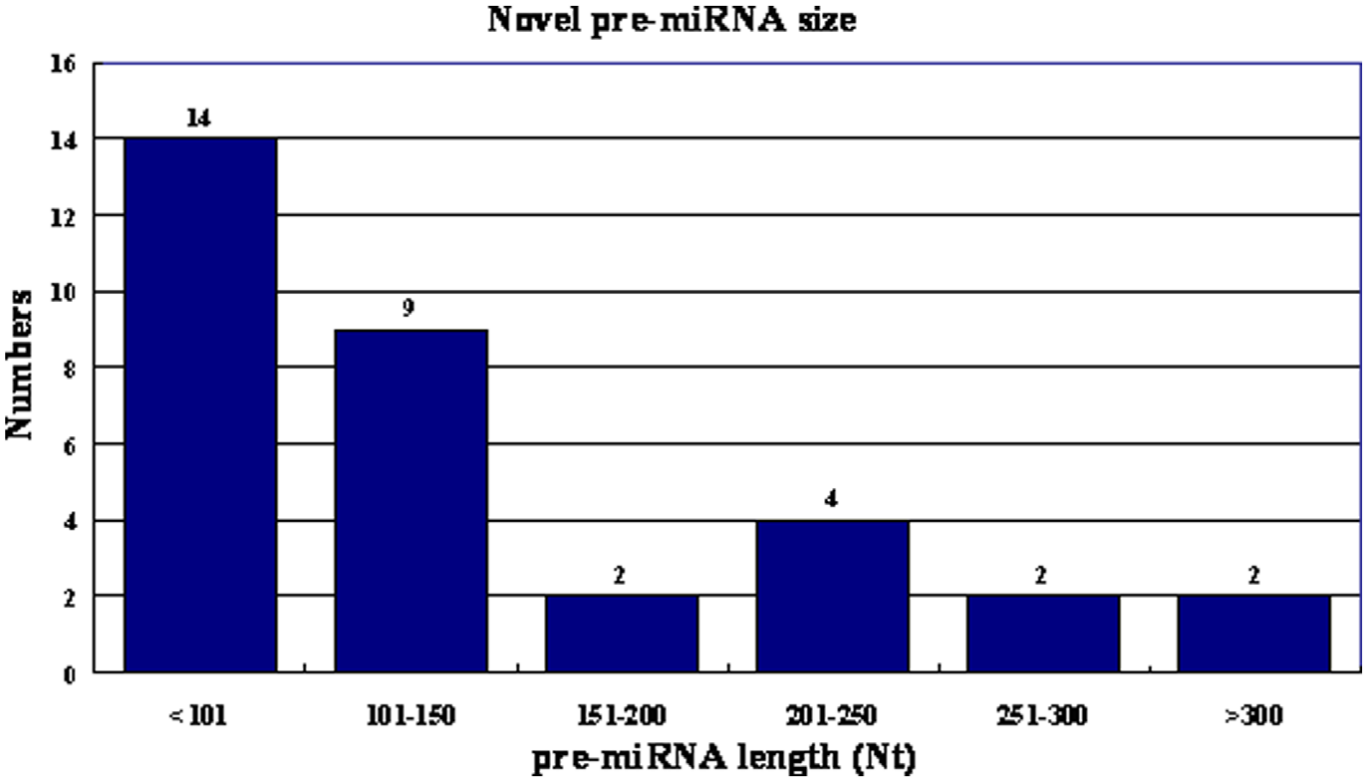
Pre-miRNA length distribution of 34 newly defined maize miRNAs.

We found 1068 small RNAs in Yu87-1 that did not match conserved miRNAs but that was located within a stem and formed stem-loop structures with their flanking sequences; these were treated as miRNA candidates. We calculated the MFEI (minimal folding free energy index) of all 1068 miRNA candidates to confirm whether they were true miRNAs. Of the 1068 miRNA candidates, 167 had MFEI greater than 0.85. These 167 candidates were searched in other samples. Thirty-four were identical between the three small RNA samples in terms of their precursor sequences, mature miRNA sequences, and chromosomal locations. These 34 sequences were most likely true novel miRNAs. The pre-miRNA length distribution of the 34 newly defined maize miRNAs is shown in Figure 1. These 34 miRNAs were classified into 20 families based on their mature miRNA sequences; their pre-miRNAs and chromosomal locations are listed in Table S2. Further more, 8 novel novel maize miRNAs were selected to validate the deep-sequencing results, and the qRT-PCR shows a perfect consistency with the Solexs results.

The target genes of these 34 newly defined miRNAs were predicted via miRU, following the same protocol used for the conserve maize miRNAs. A total of 314 unique miRNA targets were detected. These target genes were further analysed by GO (Gene Ontology, http://www.geneontology.org/) (Table S3).

### Expression Profiles of Some microRNAs and its Target Genes

**Figure 2:**
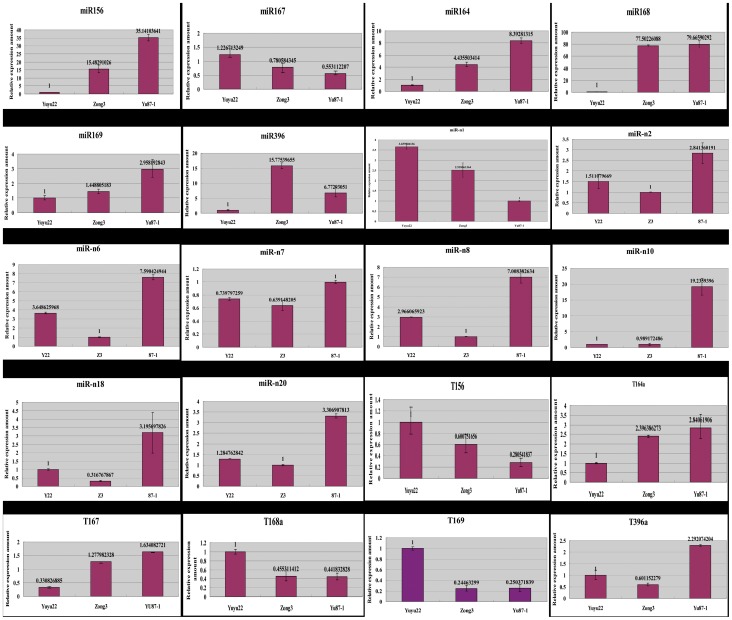
Relative expression amounts of selected miRNAs and their target genes. miR indicates the expression amount of miRNAs, and ‘T’ means the target gene.

As Zhang et al [20] have reported, the target genes of maize miRNAs were concentrated on transcriptional and translational regulators. Expression profiles were established based on stem-loop real-time RT-PCR analysis of six selected miRNAs, comprising miR156, miR164, miR167, miR168, miR169 and miR396, as well as real-time RT-PCR analysis of their target mRNAs. Each target gene of these six miRNAs, which was one of the members of a whole family, was selected as representatives of the entire group of target genes. The real-time RT-PCR data were subjected to analysis of variance (ANOVA). The results indicated that all but the target gene of miR168 showed significantly differential expression between the hybrid Yuyu22 and its parental inbred lines (Table 2). The differential expression of miRNAs showed that the sequencing data accorded well with the real-time RT-PCR reactions and the differential expression of the target genes was influenced by the corresponding miRNAs (Figure 2).

**Table 2:** ANOVA of stem-loop real-time RT-PCR analysis of miRNAs and RT-PCR analysis of target genes.

miRNA	miR156**	miR164**	miR168**	miR169*	miR396**
Target gene	T156j*	T164a*	T168a	T169a*	T396c**

Note: **^,^*significant at *P*<0.01 and *P*<0.05, respectively.

## Discussion

### Genetic Basis of Heterosis

Heterosis is not only observed in seedling and/or adult traits such as yield and plant height, but is also detected during embryo development [21], and the heterotic patterns of metabolite compositions are specific for the endosperm and embryo [22]. Some plants have evolved a strategy to activate the paternal genome immediately after fertilization. Additive and non-additive gene expression patterns in hybrid maize embryo and endosperm development were described in kernels 6 d after fertilization [22]–[23]. In addition, non-additive proteins are accumulated in the germinating embryo of four maize hybrids and their corresponding parental lines [11]. The non-additive expression pattern indicated a *trans*-regulatory mechanism, and the consistent expression of genes encoding chromatin-related proteins indicated that heterosis-related epigenetic processes are involved. In an analysis of maize genomic eQTLs, 86% of the eQTLs (differentially regulated transcripts) accumulated in a manner consistent with gene expression in the hybrid being regulated exclusively by the paternally transmitted allele, which suggested that widespread imprinting contributes to the regulation of gene expression in maize hybrids [24].

Transcriptional profiling of two *Arabidopsis* hybrids indicated that maternal or paternal transmission affected only a few genes, which suggested that the reciprocal effects observed in the analyzed crosses were minimal [25]. Based on profiling of 15 expressed genes, Guo et al [26] suggested that maternal or paternal transmission had little effect on the allele-specific transcript ratio of nearly all maize genes analyzed, and that a parent-of-origin effect was minimal in maize. In maize endosperm, genes showing maternal-like expression are more abundant than those exhibiting paternal-like expression [27]. Through detection of a large number of genes expressed in immature ear tissues of 16 maize hybrids that vary in their degree of heterosis, Guo et al [28] reported that the proportion of genes that exhibit a bias towards the expression level of the paternal parent is negatively correlated with hybrid yield and heterosis. In the present study, the expression profile of miRNAs detected in the germinating embryo in the elite maize hybrid Yuyu22 and its corresponding parental inbred lines showed a paternal dominance model. However, the influence of miRNA expression on mRNA profiling requires further investigation to dissect the genetic basis of heterosis in the miRNA regulatory mechanism.

### Differentially Expressed Heterosis-related miRNAs in Maize

Previous studies have shown that the quantity, timing, and quality of gene expression could account for phenotypic differences between inbred lines and their corresponding F_1_ hybrid [29]–[32]. Whether miRNAs are critical gene regulatory factors involved in manifestation of heterosis in maize is unclear. Previously, expression patterns of five mature miRNAs were detected in the inbred lines H99 and B73 and their F_1_ hybrid in maize. MiR167 showed a high expression level in the F_1_ hybrid, whereas the other miRNAs showed low parental expression, in kernels 10 d after fertilization [17]. Interestingly, similar results were obtained in the present study. In the maize hybrid Yuyu22, miR167 was predominantly activated, whereas miR156, miR160, miR164 and miR166 were either dominantly or predominantly repressed. The additive and non-additive expression models indicated an involvement of miRNAs in heterosis in maize seed germination.

**Figure 3:**
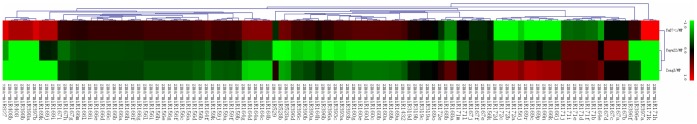
Clustering of differentially expressed microRNAs. The expression amounts derived from deep-sequencing were compared with MP (middle parental values). Green indicates down-regulation and red indicates up-regulation.

MiRNAs regulate expression of mRNAs via the splicing of target mRNA or repression of protein translation. In the present study, the target genes of the detected miRNAs were predominantly transcriptional and translational regulating factors, which might influence global changes in gene expression. The facts that most of the miRNAs were repressed in the hybrid compared to the parental inbred lines (Figure 3), and the most abundant target genes of miRNAs were transcriptional and translational regulators indicated that transcription and translation are increased in the hybrid, which might be the main mechanism for heterosis in maize seed germination. Interestingly, miR168, which targets *AGO1* and is a negative feedback regulator of *RISC* in *Arabidopsis*, was predominately repressed in the maize hybrid Yuyu22. The predominant repression of maize miR168 might cause de-repression of most other miRNA target genes, most of which are indicated to be transcription regulation factors; thus, the functional genes contributing to heterosis in hybrid might much flourish compared to the parental inbred lines.

**Figure 4:**
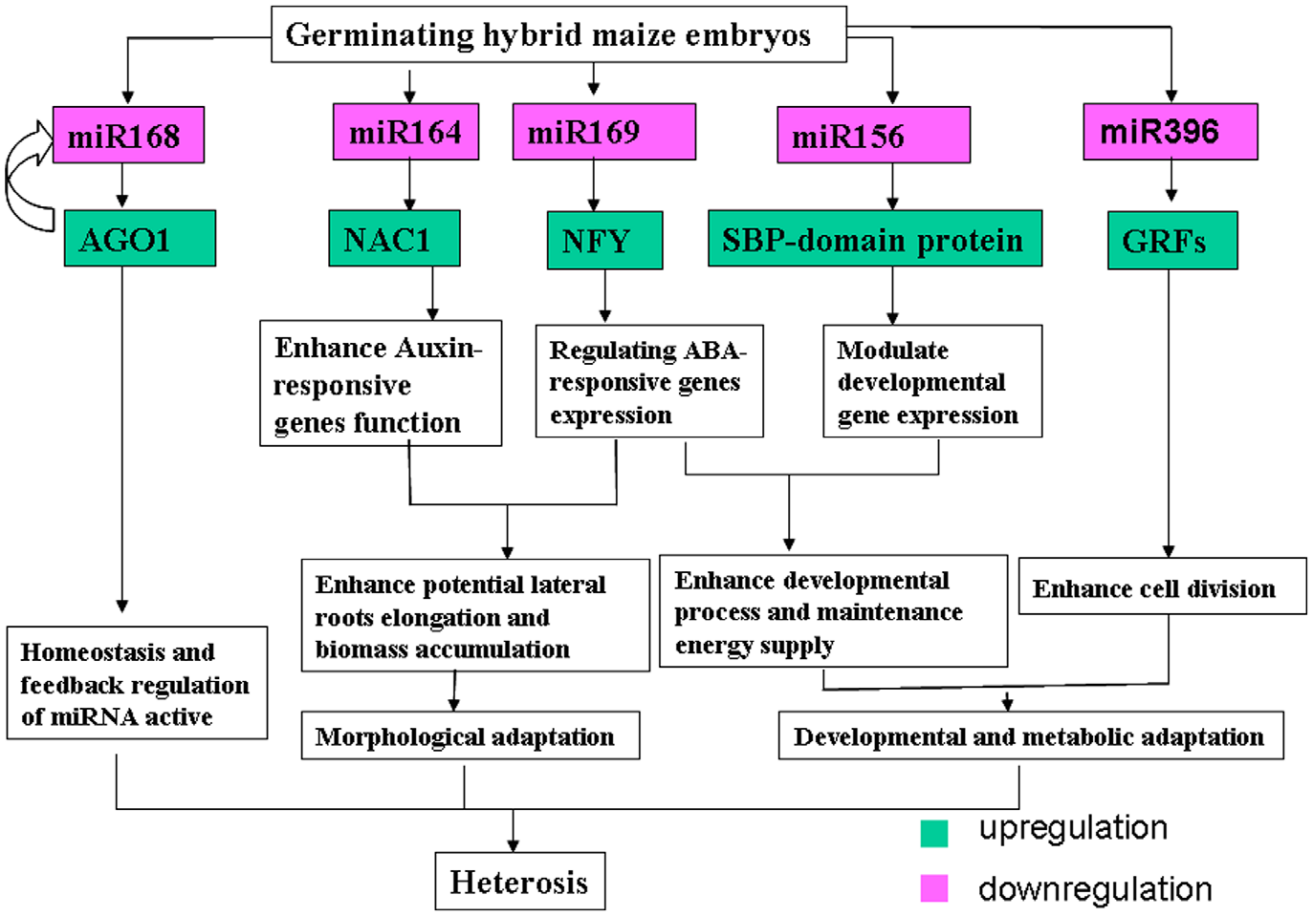
Possible microRNAs-dependent regulatory pathways that participate in heterosis during maize germination.

### miRNAs Target Transcription Regulation Factors Involved in Complex Seed Germination Regulatory Pathways

The transcription factor *NAC1* is considered to be the target gene of the maize miRNA zma-miR164. *NAC1* specially binds to the *cis*-element *IDE2* [CA(A/C)G(T/C)(T/C/A)(T/C/A) as core sequences] [33]. In many plant spaces *NAC* transcription factors are involved in plant tolerance to biotic or abiotic stresses [34]–[36]. Salt stress might cause serious inhibition of seed germination as a result of decreased levels of the phytohormone gibberellins (GAs). One of the *NAC* family members, *NTL8*, was induced by GA in the seed germination process and showed no relationship with ABA [37]. *NAC1* was induced by auxin and acts downstream of *TIR1* [38]. The down-regulation of *NAC1* transcripts caused either by auxin-induced miR164 or by ubiquitination might decrease auxin signals [39]–[40]. In the present work, zma-miR164 was repressed over dominantly in the hybrid, which indicated that up-regulation of *NAC1* transcription might occur and result in enhancement of auxin signals.

**Table 3:** Stem-loop RT and PCR primer sequences for five selected miRNAs.

Primer	Primer sequence (5′→3′)
**miR156RT**	GTCGTATCCAGTGCAGGGTCCGAGGTATTCGCACTGGATACGACGTGCTC
**miR156PCR**	CGCGCCTGACAGAAGAGAGT
**miR164RT**	GTCGTATCCAGTGCAGGGTCCGAGGTATTCGCACTGGATACGACTGCACG
**miR164PCR**	TGCTTTGGAGAAGCAGGGCA
**miR169RT**	TCGTATCCAGTGCAGGGTCCGAGGTATTCGCACTGGATACGACTCGGCA
**miR169PCR**	TGCCTCAGCCAAGGATGACT
**miR168RT**	GTCGTATCCAGTGCAGGGTCCGAGGTATTCGCACTGGATACGACGTCCCG
**miR168PCR**	TGCCTTCGCTTGGTGCAGAT
**miR396RT**	TCGTATCCAGTGCAGGGTCCGAGGTATTCGCACTGGATACGACCAGTTC
**miR396PCR**	CGGTCTTCCACAGCTTTCTT
**miRn1RT**	TCGTATCCAGTGCAGGGTCCGAGGTATTCGCACTGGATACGACTAGCCC
**miRn1PCR**	CTAGTTGGAGGGGATTGAGG
**miRn2RT**	TCGTATCCAGTGCAGGGTCCGAGGTATTCGCACTGGATACGACCTTATC
**miRn2PCR**	CCTAGTAACTAGCCGTCGGA
**miRn6RT**	GTCGTATCCAGTGCAGGGTCCGAGGTATTCGCACTGGATACGACCGGGGA
**miRn6PCR**	CTAGTCGGACCAGGCTTCAT
**miRn7RT**	GTCGTATCCAGTGCAGGGTCCGAGGTATTCGCACTGGATACGACCGTCTG
**miRn7PCR**	CGGCGATGCAGAACAATTTA
**miRn8RT**	GTCGTATCCAGTGCAGGGTCCGAGGTATTCGCACTGGATACGACACTCCC
**miRn8PCR**	GCGCAAGACTTAGGAACGGA
**miRn10RT**	GTCGTATCCAGTGCAGGGTCCGAGGTATTCGCACTGGATACGACGCAGCT
**miRn10PCR**	CAACAGTGAGACCCTGCAGA
**miRn18RT**	GTCGTATCCAGTGCAGGGTCCGAGGTATTCGCACTGGATACGACCTTATT
**miRn18PCR**	GCAATAACTAGCCGTCGGAG
**miRn20RT**	GTCGTATCCAGTGCAGGGTCCGAGGTATTCGCACTGGATACGACTAACCA
**miRn20PCR**	CCGGCATGCACTAGAGCTAA
**Universal Reverse**	GTGCAGGGTCCGAGGT

**Table 4:** Accessions (TIGR Maize Gene Index 15) of target genes and primers used for RT-PCR.

miRNA target	Gene	Accession no.	Primer (sense)	Primer (antisense)
T156j	*SBP5*	TC272836	GCTGGCAACACGATGGAC	CAAACTTTCAGCCTCCTCCA
T164a	*NAC1*	TC258020	TACAAGAGTAGGGCAACA	AGGCTGGTCAAAGGAGAT
T168a	*AGO1*	TC249282	GCAATCTCCGCCGCTTCGT	TCTTCCGCTGCCGCTCCTT
T169a	*NTF-Y*	TC271961	CAAGCAAGGTGGGAAGAG	GGAGTCGTAATCAGGCATC
T396c	*GRF*	TC261753	ACAAACTACCTGCCTTACA	GATTCAATAGCCAACCATA
Internal control	*Actin1*	BT039000	AATGACGCAGATTATGTTTG	TTAGGTGGTCGGTGAGGT

Nuclear factor Y (NFY) transcription factors bind promoters containing a conserved CCAAT *cis*-element in both the plant and animal kingdoms. In animals, NFY transcription factors are encoded by few genes, whereas in *Arabidopsis* 36 genes are known to encode NFY transcription factors. These factors combine with each other to establish a complex that might function in regulation of gene expression [41]. In imbibed seeds, transcription induced by ABA and blue light was regulated by a series of transcription factors including NFY, which might influence seed germination ratios [42]. LEC1, one of the NFY transcription factors in *Arabidopsis*, was a key factor in seed germination [43]. NFYA5 was a target gene of miR169 and is post-transcriptionally regulated by miR169 [44]. In the present study, the maize miRNA zma-miR169 was repressed over dominantly, which might influence ABA-responsive transcription and result in enhancement of the seed germination capability of hybrid seeds.

SQUAMOSA promoter-binding-like (SPL) family transcription factors are the target of the maize miRNA zma-miR156, which specially binds to the *cis*-element GTAC [45]. SPL transcription factors have multiple functions. Regulating omics studies have reported that in addition to metabolic functions, SPL transcription factors influence developmental processes by regulating other transcription factor families [46]. Most studies of SPL transcription factors in plants have focused on plant development, especially on the transition from nutritional to reproductive development and on meristem formation [47]–[48]. In the present study, zma-miR156 was over dominantly repressed, which might cause over-expression of SPL transcription factors and result in enhancement of seed germination by influencing the expression of other transcription factors.

Studies on the model plants rice and *Arabidopsis* indicate that growth regulating factors (*GRFs*) were the conserved target genes of miR396 in dicotyledon and monocotyledon plants [49]–[50]. *GRFs* are strongly expressed in actively growing and developing tissues, such as shoot tips, flower buds, and roots, but are weakly expressed in mature stem and leaf tissues. The members of *GRF* families are functionally substituted [51]. It is clear that miR396 plays an important role in plant leaf growth and development, most likely by repression of *GRF* gene expression. Ectopic over-expression of miR396 represses expression of not only six *GRF* genes but also *GIF1*, which encodes a *GRF*-interacting transcription co-activator with a role in cell proliferation in the leaf [49]. In maize, the targets of miR396 were also *GRF* genes, and our study showed that miR396 was over dominantly repressed. Since GRFs might regulate cell division, it is possible that repression of miR396, which leads to inducement of GRFs, enhances cell division in Yuyu22 compared to that in the parental inbred lines.

As of the conserved miRNA ones, the target genes of novel miRNAs were also fasten on the transcriptional, translational regulation and signal transduction (Figure 4). In this work, 8 novel miRNAs were selected to validate the expression results. The interaction of novel miRNAs and their predicted target genes needs further attention.

## Materials and Methods

### Plant Materials, RNA Extraction and Deep Sequencing

To obtain the heterosis related miRNAs, next generation deep-sequencing technique Solexa was performed. In this study the hybrid maize Yuyu22, which is an elite hybrid widely cultivated in China that exhibits high heterosis for grain yield, and its parental inbred lines Yu87-1 and Zong3 were utilized. The dent inbred line Zong3 was selected from a synthetic population of Chinese domestic germplasm, and the flint inbred line Yu87-1 was selected from exotic germplasm [18].

Twenty uniform seeds of the hybrid and each parental line were surface-sterilized in 70% (v/v) ethanol for 15 min, and then rinsed several times with sterile distilled water. To obtain embryo tissues for each line, the seeds were submerged in sterile distilled water and incubated at 28°C in the dark for 24 h. The embryo tissues were peeled from the seeds, and each embryo tissue material was mixed together for immediate RNA extraction. Total RNA from the combined samples was extracted with TRIzol reagent (Invitrogen, 15596026) following the manufacturer’s protocol. A sample of 20 ng RNA was used for miRNA extraction and Solexa technology (LF10096, BGI, China).

### Bioinformatic Analysis

The deep-sequencing raw data were pre-processed to eliminate low-quality tags, and the appropriate tags of miRNAs were obtained. Ordinarily, the small RNAs were 18 nt to 30 nt in length; the RNA tags with appropriate length were processed using PHRED and CROSSMATCH programs as reported. In detail, the tRNA, rRNA, snRNA, snoRNA and the small sequences containing polyA tail were removed first and the remaining sequences were compared against maize B73 sequence database (maizeseuqence.org). Then, the unique small RNA sequences were blasted against the miRNA database, miRBase18.0, to identify conserved miRNAs in maize. The expression amounts of all given maize conserved miRNA in the hybrid and its parental inbred lines were obtained via the deep-sequencing reads. In order to get the comparable expression amounts, ratio between the sequence reads and the total sample reads were calculated and measured by RPM (Reads per million). For each conserved miRNA, mid-parental value was calculated based on the comparable expression amounts. The miRNAs with distinct expression amounts between the hybrid and its parental inbred lines were selected as candidate embryo heterosis-related miRNAs. Briefly, a difference between expression amounts in the hybrid and mid-parental value of below 10% was used to estimate additive influence, a difference between the hybrid and parental lines of below 10% was used to estimate dominance influence, and other differences in expression amounts represented over dominant miRNAs.

To identify novel miRNAs, prediction software Mireap (http://sourceforge.net/projects/mireap/) was used to process the unannotated small RNA reads that mapped to the maize genome. A small RNA is considered a potential miRNA candidate only if it meets the following strict criteria: 1) its initial transcript can fold into an appropriate stem-loop hairpin secondary structure; 2) the small RNA sequence comprises one arm of the hairpin structure; 3) there are no more than six mismatches between the predicted mature miRNA sequence and its complementary miRNA* sequence on the other arm of the hairpin; 4) no loops or breaks exist in the miRNA or miRNA* sequences; and 5) the predicted secondary structure has a high MFEI and negative minimal folding free energy. The stem-loop structure is characteristic of miRNA precursors, yet it is not sufficient by itself to identify miRNAs. Thus, a new criterion that combined several parameters, the MFEI [52], was developed. More than 90% of miRNA precursors are reported to have an MFEI greater than 0.85, while other RNAs have MFEIs lower than 0.85 [53], indicating that MFEI is an ideal criterion to distinguish miRNA precursors from other types of RNAs. Further more, the miRNAs detected in all three samples were conformed to newly detected maize miRNAs.

### Validation of Deep Sequencing

Six candidate heterosis-related conserved maize miRNAs and 8 novel miRNAs were selected to validate the deep-sequencing results. Expression profiles of each of the target genes were analyzed in the hybrid and parental inbred lines. Stem-loop reverse transcription reactions were used for the mature miRNAs as well as RT reactions for target genes, both followed by real-time PCR reactions. *Actin1* served as an internal control in the real-time RT-PCR reactions, and the 2^−ΔΔCt^ method was used to calculate relative expression amounts. The PCR primers of selected mature miRNAs and their target genes are listed in Tables 3 and 4. Each real-time RT-PCR reaction was done in technical triplicates. Data analyses were performed using SAS 8.0 statistical software with the PROC MIXED procedure.

## Supporting Information

Table S1 Differential expression of maize microRNAs in Yuyu22 and its parental inbred lines (the values show the detected reads of each microRNAs in solexa, measuring by RPM, reads per million).
(DOC)

Table S2 Predicted novel miRNAs (chromosome location, pre-miRNA length, mature miRNA sequence, MFEI and detected reads of each miRNAs were listed).
(XLS)

Table S3 Novel miRNA target genes (target gene ID and Gene Ontology were listed).
(XLS)
