# Supplementary material for: MicroRNA Transcriptomic Analysis of Heterosis during Maize Seed Germination
Source: PLoS One. 2012 Jun 27;7(6):e39578. doi: 10.1371/journal.pone.0039578 (PMC3384671; doi:10.1371/journal.pone.0039578)
Supplement: Table S1 — Differential expression of maize microRNAs in Yuyu22 and its parental inbred lines (the values show the detected reads of each microRNAs in solexa, measuring by RPM, reads per million). (DOC) [file pone.0039578.s001.doc]

Supplement table 1: Differential expression of maize microRNAs in Yuyu22 and its parental inbred lines (the values show the detected reads of each microRNAs in solexa, measuring by RPM, reads per million.).

| Expression trends | miR | miR sequence | Yu87-1 | Zong3 | Yuyu22 |
| --- | --- | --- | --- | --- | --- |
| -- | zma-miR1432 | CUCAGGAGAGAUGACACCGAC | 3.8005 | 2.4333 | 1.1745 |
| - | zma-miR156a | UGACAGAAGAGAGUGAGCAC | 40696.1 | 27127.245 | 28632.936 |
| - | zma-miR156b | UGACAGAAGAGAGUGAGCAC | 44812.72 | 28231.36 | 30452.118 |
| - | zma-miR156c | UGACAGAAGAGAGUGAGCAC | 40695.99 | 27119.945 | 28631.49 |
| - | zma-miR156d | UGACAGAAGAGAGUGAGCAC | 44824.53 | 28234.63 | 30456.093 |
| - | zma-miR156e | UGACAGAAGAGAGUGAGCAC | 40662.61 | 27050.139 | 28596.525 |
| - | zma-miR156f | UGACAGAAGAGAGUGAGCAC | 40630.05 | 27080.48 | 28600.591 |
| - | zma-miR156g | UGACAGAAGAGAGUGAGCAC | 40629.95 | 27080.48 | 28600.41 |
| - | zma-miR156h | UGACAGAAGAGAGUGAGCAC | 40662.61 | 27050.139 | 28596.525 |
| - | zma-miR156i | UGACAGAAGAGAGUGAGCAC | 40662.71 | 27050.139 | 28596.525 |
| -- | zma-miR156j | UGACAGAAGAGAGAGAGCACA | 18.5915 | 47.2215 | 26.4723 |
| +-/- | zma-miR156k | UGACAGAAGAGAGCGAGCAC | 574.6919 | 649.4661 | 561.5205 |
| - | zma-miR156l | UGACAGAAGAGAGUGAGCAC | 40660.15 | 27041.395 | 28594.989 |
| +- | zma-miR159a | UUUGGAUUGAAGGGAGCUCUG | 23.6245 | 15.1322 | 18.3409 |
| +- | zma-miR159b | UUUGGAUUGAAGGGAGCUCUG | 23.4191 | 14.9801 | 17.8891 |
| +- | zma-miR159f | UUUGGAUUGAAGGGAGCUCUG | 23.4191 | 14.752 | 18.3409 |
| +- | zma-MIR159j | UUUGGAUUGAAGGGAGCUCUG | 23.2137 | 14.5999 | 17.8891 |
| +- | zma-MIR159k | UUUGGAUUGAAGGGAGCUCUG | 23.2137 | 14.5999 | 17.8891 |
| -- | zma-miR160a | UGCCUGGCUCCCUGUAUGCCA | 4.1086 | 2.3573 | 1.5359 |
| -- | [zma-MIR160b](http://www.mirbase.org/cgi-bin/mirna_entry.pl?acc=MI0001468) | UGCCUGGCUCCCUGUAUGCCA | 4.0059 | 2.2812 | 1.5359 |
| -- | [zma-MIR160c](http://www.mirbase.org/cgi-bin/mirna_entry.pl?acc=MI0001466) | UGCCUGGCUCCCUGUAUGCCA | 4.0059 | 2.2812 | 1.5359 |
| -- | [zma-MIR160d](http://www.mirbase.org/cgi-bin/mirna_entry.pl?acc=MI0001467) | UGCCUGGCUCCCUGUAUGCCA | 4.0059 | 2.2812 | 1.5359 |
| -- | zma-MIR160e | UGCCUGGCUCCCUGUAUGCCA | 4.0059 | 2.2812 | 1.5359 |
| -- | zma-miR160g | UGCCUGGCUCCCUGUAUGCCA | 4.0059 | 2.2812 | 1.5359 |
| D | [zma-MIR164a](http://www.mirbase.org/cgi-bin/mirna_entry.pl?acc=MI0001469) | UGGAGAAGCAGGGCACGUGCA | 93.779 | 36.0434 | 50.0535 |
| D | zma-MIR164b | UGGAGAAGCAGGGCACGUGCA | 93.779 | 36.0434 | 50.1438 |
| D | zma-MIR164c | UGGAGAAGCAGGGCACGUGCA | 93.779 | 36.0434 | 50.1438 |
| D | zma-MIR164d | UGGAGAAGCAGGGCACGUGCA | 93.779 | 36.0434 | 50.0535 |
| +- | zma-MIR164e | UGGAGAAGCAGGACACGUGAG | 1470.575 | 3123.6881 | 2342.2134 |
| D | zma-MIR164f | UGGAGAAGCAGGGCACGUGCU | 4.2113 | 2.3573 | 2.6201 |
| D | zma-MIR164g | UGGAGAAGCAGGGCACGUGCA | 89.465 | 35.5111 | 49.15 |
| -- | zma-MIR164h | UGGAGAAGCAGGGCACGUGUG | 2.0543 | 1.6729 | 1.0842 |
| - | zma-MIR166a | UCGGACCAGGCUUCAUUCCCC | 28611.24 | 20565.896 | 20245.094 |
| - | zma-MIR166b | UCGGACCAGGCUUCAUUCCC | 28609.59 | 20563.995 | 20243.287 |
| - | zma-MIR166c | UCGGACCAGGCUUCAUUCCC | 28609.59 | 20563.995 | 20243.287 |
| - | zma-MIR166d | UCGGACCAGGCUUCAUUCCC | 28609.59 | 20563.995 | 20243.287 |
| - | zma-MIR166e | UCGGACCAGGCUUCAUUCCC | 28609.59 | 20564.071 | 20243.377 |
| - | zma-MIR166f | UCGGACCAGGCUUCAUUCCC | 28551.04 | 20635.853 | 20341.316 |
| - | zma-MIR166g | UCGGACCAGGCUUCAUUCCC | 28550.22 | 20632.127 | 20336.256 |
| - | zma-MIR166h | UCGGACCAGGCUUCAUUCCC | 28551.04 | 20635.853 | 20341.316 |
| - | zma-MIR166i | UCGGACCAGGCUUCAUUCCC | 28607.23 | 20562.626 | 20241.57 |
| D | zma-MIR166j | UCGGACCAGGCUUCAAUCCCU | 41.1888 | 144.4779 | 80.9529 |
| D | zma-MIR166k | UCGGACCAGGCUUCAAUCCCU | 41.1888 | 144.4019 | 80.9529 |
| - | zma-MIR166l | UCGGACCAGGCUUCAUUCCUC | 2687.545 | 2117.5896 | 1985.6954 |
| - | zma-MIR166m | UCGGACCAGGCUUCAUUCCUC | 2628.689 | 2068.7713 | 1936.9068 |
| D | zma-MIR166n | UCGGACCAGGCUUCAAUCCCU | 41.1888 | 144.4019 | 80.9529 |
| ++ | zma-MIR167a | UGAAGCUGCCAGCAUGAUCUA | 783.4093 | 836.2228 | 1217.0945 |
| ++ | zma-MIR167b | UGAAGCUGCCAGCAUGAUCUA | 782.6903 | 835.6905 | 1216.2813 |
| ++ | zma-MIR167c | UGAAGCUGCCAGCAUGAUCUA | 783.4093 | 836.2228 | 1217.0945 |
| ++ | zma-MIR167d | UGAAGCUGCCAGCAUGAUCUA | 783.4093 | 836.2228 | 1217.0945 |
| - | zma-MIR167e | UGAAGCUGCCAGCAUGAUCUG | 350.7726 | 441.1138 | 355.4339 |
| - | zma-MIR167f | UGAAGCUGCCAGCAUGAUCUG | 350.8754 | 441.6461 | 355.5242 |
| - | zma-MIR167g | UGAAGCUGCCAGCAUGAUCUG | 347.1776 | 265.9914 | 247.0148 |
| D | zma-MIR167h | UGAAGCUGCCAGCAUGAUCUG | 425.2412 | 306.8254 | 271.4091 |
| D | zma-MIR167i | UGAAGCUGCCAGCAUGAUCUG | 425.7548 | 306.9015 | 271.4091 |
| - | zma-MIR167j | UGAAGCUGCCAGCAUGAUCUG | 350.8754 | 442.5586 | 356.247 |
| -- | zma-MIR168a | UCGCUUGGUGCAGAUCGGGAC | 5544.159 | 10139.23 | 4047.9165 |
| -- | zma-MIR168b | UCGCUUGGUGCAGAUCGGGAC | 5544.159 | 10139.23 | 4047.9165 |
| -- | zma-MIR169a | CAGCCAAGGAUGACUUGCCGA | 92.4437 | 54.2172 | 29.2732 |
| -- | zma-MIR169b | CAGCCAAGGAUGACUUGCCGA | 90.3894 | 52.8485 | 28.9118 |
| +- | zma-MIR169c | CAGCCAAGGAUGACUUGCCGG | 17.3589 | 74.6723 | 42.1027 |
| +- | zma-MIR169i | UAGCCAAGGAUGACUUGCCUG | 7.7036 | 1.6729 | 4.2464 |
| +- | zma-MIR169j | UAGCCAAGGAUGACUUGCCUG | 7.3955 | 1.5969 | 4.1561 |
| +- | zma-MIR169k | UAGCCAAGGAUGACUUGCCUG | 7.7036 | 1.6729 | 4.3368 |
| -- | zma-MIR169o | UAGCCAAGAAUGACUUGCCUA | 103.6397 | 106.1532 | 47.8851 |
| +- | zma-MIR169p | UAGCCAAGGAUGACUUGCCGG | 0.719 | 2.6614 | 1.6263 |
| +- | zma-MIR169r | CAGCCAAGGAUGACUUGCCGG | 17.2562 | 74.0639 | 42.0124 |
| + | zma-MIR171b | UUGAGCCGUGCCAAUAUCAC | 5.7521 | 9.8853 | 10.0288 |
| + | zma-MIR171d | UGAUUGAGCCGUGCCAAUAUC | 5.9575 | 9.7332 | 10.1191 |
| + | zma-MIR171e | UGAUUGAGCCGUGCCAAUAUC | 5.9575 | 9.7332 | 10.1191 |
| + | zma-MIR171f | UUGAGCCGUGCCAAUAUCACA | 5.7521 | 9.8093 | 10.0288 |
| D | zma-MIR171h | GUGAGCCGAACCAAUAUCACU | 1.0272 | 0.01 | 0.1807 |
| + | zma-MIR171i | UGAUUGAGCCGUGCCAAUAUC | 5.8548 | 9.8093 | 10.1191 |
| + | zma-MIR171j | UGAUUGAGCCGUGCCAAUAUC | 5.9575 | 9.8853 | 10.2095 |
| D | zma-MIR171k | GUGAGCCGAACCAAUAUCACU | 1.0272 | 0.01 | 0.1807 |
| -- | zma-MIR171l | GGAUUGAGCCGCGUCAAUAUC | 4.1086 | 4.1062 | 3.2526 |
| -- | zma-MIR171m | GGAUUGAGCCGCGUCAAUAUC | 4.1086 | 4.1062 | 3.2526 |
| D | zma-MIR172a | AGAAUCUUGAUGAUGCUGCA | 2.0543 | 6.9197 | 3.4333 |
| D | zma-MIR172b | AGAAUCUUGAUGAUGCUGCA | 2.0543 | 6.9197 | 3.4333 |
| D | zma-MIR172c | AGAAUCUUGAUGAUGCUGCA | 2.0543 | 6.9197 | 3.4333 |
| D | zma-MIR172d | AGAAUCUUGAUGAUGCUGCA | 2.0543 | 6.9197 | 3.4333 |
| -- | zma-MIR172e | GGAAUCUUGAUGAUGCUGCAU | 0.8217 | 1.0646 | 0.5421 |
| -- | zma-MIR319a | UUGGACUGAAGGGUGCUCCC | 51.7685 | 48.6662 | 19.6058 |
| -- | zma-MIR319b | UUGGACUGAAGGGUGCUCCC | 61.6292 | 52.012 | 22.768 |
| -- | zma-MIR319c | UUGGACUGAAGGGUGCUCCC | 51.6658 | 48.6662 | 19.6058 |
| -- | zma-MIR319d | UUGGACUGAAGGGUGCUCCC | 61.6292 | 52.012 | 22.768 |
| -- | zma-MIR390a | AAGCUCAGGAGGGAUAGCGCC | 24.4462 | 17.1853 | 11.5647 |
| -- | zma-MIR390b | AAGCUCAGGAGGGAUAGCGCC | 24.4462 | 17.1853 | 11.5647 |
| -- | zma-MIR393a | UCCAAAGGGAUCGCAUUGAUCU | 4.9303 | 3.0416 | 2.078 |
| -- | zma-MIR393b | UCCAAAGGGAUCGCAUUGAUCC | 2.3625 | 1.4448 | 0.9035 |
| -- | zma-MIR393c | UCCAAAGGGAUCGCAUUGAUCU | 4.9303 | 3.0416 | 2.078 |
| -- | zma-MIR396a | UUCCACAGCUUUCUUGAACUG | 9.2444 | 6.8437 | 3.7947 |
| -- | zma-MIR396b | UUCCACAGCUUUCUUGAACUG | 9.2444 | 6.8437 | 3.7947 |
| -- | zma-MIR396c | UUCCACAGGCUUUCUUGAACUG | 23.5218 | 15.2842 | 11.2033 |
| -- | zma-MIR396d | UUCCACAGGCUUUCUUGAACUG | 23.5218 | 15.2842 | 11.2033 |
| -- | zma-MIR396e | UUCCACAGCUUUCUUGAACUU | 3.0815 | 2.2052 | 0.271 |
| -- | zma-MIR396f | UUCCACAGCUUUCUUGAACUU | 3.0815 | 2.2052 | 0.271 |
| D | zma-MIR397a | UCAUUGAGCGCAGCGUUGAUG | 42.6268 | 0.4562 | 5.3306 |
| D | zma-MIR397b | UCAUUGAGCGCAGCGUUGAUG | 92.2383 | 9.9614 | 14.1848 |
| - | zma-MIR398a | UGUGUUCUCAGGUCGCCCCCG | 55.6717 | 13.8395 | 14.998 |
| - | zma-MIR398b | UGUGUUCUCAGGUCGCCCCCG | 56.1853 | 13.8395 | 14.998 |
| -- | zma-MIR408 | CUGCACUGCCUCUUCCCUGGC | 116.3764 | 29.8081 | 20.1479 |
| -- | zma-MIR408b | CUGCACUGCCUCUUCCCUGGC | 115.9655 | 29.656 | 20.1479 |
| -- | zma-MIR528a | UGGAAGGGGCAUGCAGAGGAG | 54724.95 | 26262.431 | 20423.985 |
| -- | zma-MIR528b | UGGAAGGGGCAUGCAGAGGAG | 57298.79 | 26399.609 | 20815.378 |
| -- | zma-MIR529 | AGAAGAGAGAGAGUACAGCCU | 13.8666 | 17.4894 | 3.9754 |
| -- | zma-MIR827 | UUAGAUGACCAUCAGCAAACA | 129.3185 | 10.2655 | 45.1746 |
